# ERAS protocol vs. conventional care in elective laparoscopic colorectal cancer surgery in Hatyai Hospital

**DOI:** 10.3389/fsurg.2025.1710191

**Published:** 2025-12-11

**Authors:** Kamales Prasitvarakul, Danusorn Paekaittiwong, Hathaitip Tumviriyakul, Araya Khaimook

**Affiliations:** 1 Department of Surgery, Hatyai Hospital, Hatyai, Songkhla, Thailand; 2 Department of Family Medicine, Hatyai Hospital, Hatyai, Songkhla, Thailand; 3 Information and Communication Technology Center, Office of Permanent Secretary, Ministry of Public Health, Nonthaburi, Thailand

**Keywords:** ERAS, laparoscopic colorectal surgery, colorectal cancer, enhanced recovery after surgery, hospital stay, ERAS protocol compliance

## Abstract

**Background:**

Enhanced recovery after surgery (ERAS) programs and laparoscopic techniques independently reduce hospital stays and postoperative complications in patients with colorectal cancer. However, evidence regarding whether the combination of ERAS protocols with laparoscopic surgery further improves postoperative outcomes remains limited.

**Objective:**

The aim of the study was to compare the postoperative hospital stay (POHS) and perioperative outcomes between patients undergoing elective laparoscopic colorectal cancer surgery under the ERAS protocol and conventional care.

**Methods:**

This ambispective cohort study included patients who underwent elective laparoscopic colorectal surgery for colorectal adenocarcinoma at Hatyai Hospital between June 2019 and May 2023. Patients were divided into a conventional group and an ERAS group. The primary outcome was POHS. Secondary outcomes included postoperative complications and 30-day readmission.

**Results:**

A total of 140 patients were included (70 ERAS, 70 conventional). Baseline characteristics were similar between groups, though the ERAS group had more preoperative chemoradiotherapy (CCRT) (52.9% vs. 39.4%; *p* = 0.002) and diverting stomas (38.6% vs. 21.4%; *p* = 0.042). The ERAS group had significantly shorter POHS (median 5.0 vs. 5.5 days; *p* < 0.001), earlier oral intake (3 vs. 4 days; *p* = 0.001), and earlier Jackson–Pratt (JP) drain removal (*p* = 0.006). There were no significant differences in postoperative complications, readmission, or mortality. Multivariate analysis identified early JP drain removal, early discontinuation of intravenous fluids, nasogastric tube avoidance, and multimodal analgesia as significant predictors of POHS ≤5 days.

**Conclusion:**

ERAS implementation in elective laparoscopic colorectal cancer surgery significantly reduces hospital stay without increasing complication or readmission rates. These findings support the safety and effectiveness of ERAS in a regional Thai hospital setting and advocate for broader protocol adoption.

## Introduction

Laparoscopic surgery is widely recognized as a standard approach for colorectal cancer due to its association with reduced postoperative pain, shorter hospital stays, and lower rates of surgical site infections (SSI), while offering long-term oncological outcomes comparable to open surgery (1–3). However, optimal surgical outcomes depend not only on the operative technique but also on comprehensive perioperative care.

Enhanced recovery after surgery (ERAS) programs, which integrate evidence-based preoperative, intra-operative, and postoperative care strategies, aim to improve recovery, minimize complications, shorten length of hospital stay (LOS), and enhance overall functional outcomes (4, 5). Key components of ERAS include early mobilization, multimodal analgesia, early enteral nutrition, and minimal use of tubes and drains (6).

Since February 2022, Hatyai Hospital has adopted ERAS protocols for patients undergoing elective laparoscopic colorectal cancer surgeries. The hospital's objective is to standardize patient care, optimize recovery, and reduce hospital burden while maintaining clinical safety (7, 8). However, given the limited number of ERAS studies in Southeast Asia, particularly in regional Thai settings, there is a need to evaluate the protocol's impact in this context (9).

This study was conducted to compare postoperative outcomes, especially length of hospital stay, between patients managed under the ERAS protocol and those receiving conventional care. Findings from this investigation are expected to provide evidence-based insights into the effectiveness and safety of ERAS in the regional hospital setting of Thailand and support further programmatic implementation.

## Methods

### Study design and setting

This ambispective cohort study was conducted at Hatyai Hospital, a referral center in southern Thailand. The study period was between June 2019 and May 2023. In February 2022, an enhanced recovery protocol for elective colonic resection was implemented at Hatyai Hospital (the ERAS group). Patients operated on between June 2019 and January 2022 were added to the conventional group. All procedures were performed by two highly experienced surgeons, each with more than 200 cases of laparoscopic colorectal surgery.

### Patient selection

**Figure 1:**
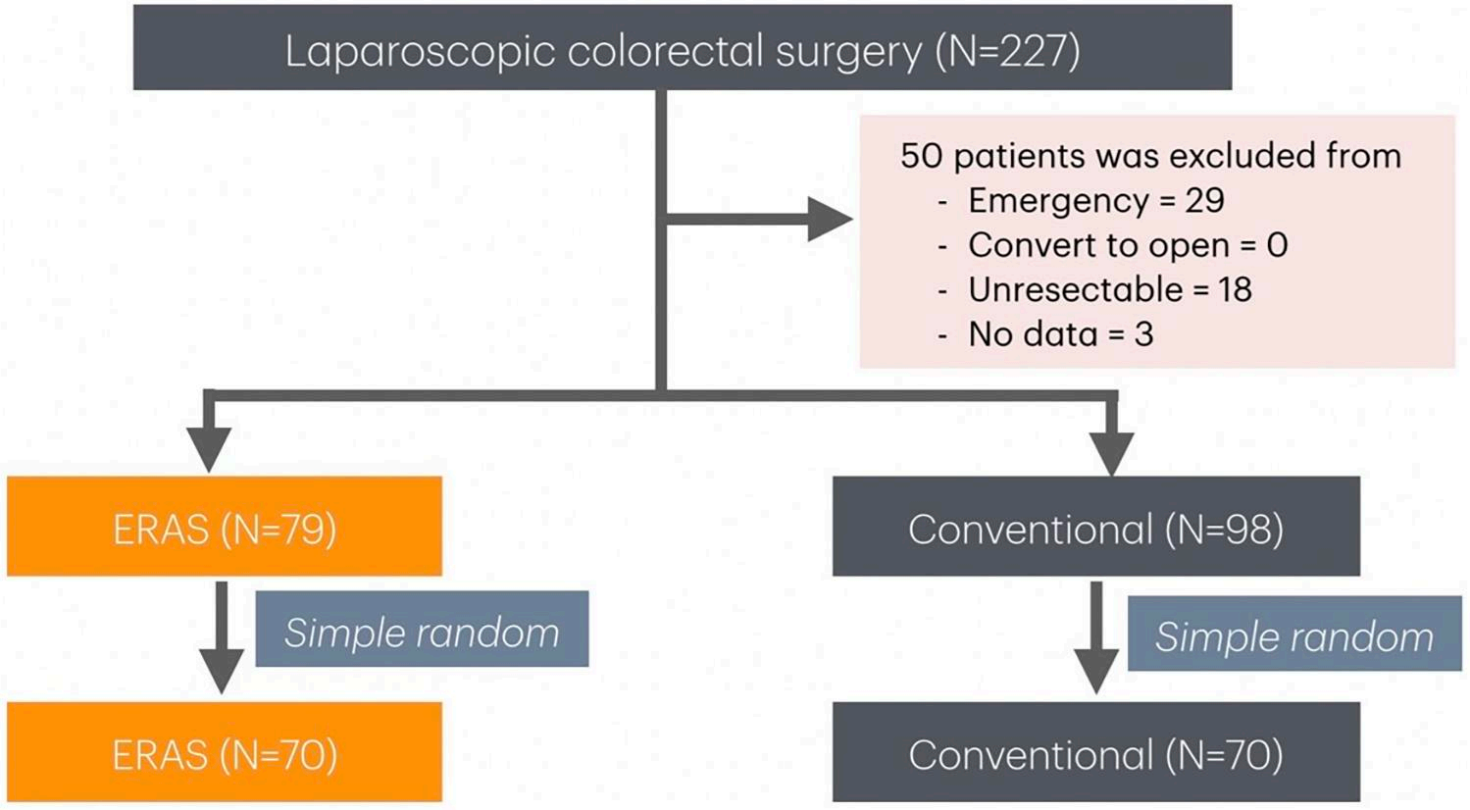
Study flow diagram.

Patients aged ≥18 years with a histologically confirmed diagnosis of colorectal adenocarcinoma who underwent elective laparoscopic colorectal resection were included. Patients were excluded if they underwent emergency surgery (due to bowel obstruction or perforation), un-resectable disease, inflammatory bowel disease, familial polyposis, previous malignancy, or severe co-morbid illness. Cases converted from laparoscopic to open surgery were also excluded. Patients operated on between June 2019 and January 2022 served as a conventional group. In February 2022, an ERAS protocol for elective laparoscopic colorectal cancer resection was implemented at Hatyai Hospital, and most patients formed the ERAS group. Informed consent was obtained from all patients before surgery and the study protocol was approved by the Human Research Ethics Committee of Hatyai Hospital, Songkhla Province (ID: HYH EC 042-66-01). A total of 227 patients were included; of them, 50 were excluded from the study as described in the study flow diagram (Figure 1). This left 177 eligible patients: 79 in the ERAS group and 98 in the conventional group. To ensure comparability, simple random sampling by computer was used to select 70 patients from each group for analysis.

### Data collection and follow-up

Pre-, peri-, and postoperative data for 30 days were recorded routinely into a prospective database. Demographic information included age, sex, body mass index (BMI), American Society Anesthesiologist (ASA) score, comorbidities (diabetes, hypertension, cardiovascular disease, pulmonary disease, and other), disease location (right-side colon cancer as a tumor from the cecum to the proximal two-thirds of the transverse colon, left-side colon cancer as a tumor from the distal one-third of the transverse colon to the sigmoid colon, and rectum cancer as a tumor from the rectosigmoid junction, below peritoneal reflection, to the anal verge). American Joint Committee on Cancer (AJCC) staging was also recorded, along with surgical information, including main operative procedure, combined procedures, duration, and estimated blood loss.

All patients were discharged once they met the following predefined criteria: (1) adequate pain control with oral medication; (2) ability to tolerate a soft diet; (3) absence of nausea; (4) passage of flatus/stool; (5) ability to mobilize as before surgery; and (6) patient agreement to discharge.

Both groups attended a follow-up outpatient visit 7–14 days after discharge. Any hospitalization within 30 days postoperatively, after discharge, was considered a readmission. Postoperative hospital stay (POHS) was defined as the number of nights spent in the hospital after surgery. To assess the impact of complications on postoperative recovery and hospital stay, all adverse events occurring during the postoperative period were recorded.

### Enhanced recovery after surgery protocol

The establishment of an ERAS protocol in our hospital was initiated by the multidisciplinary Department of Minimal Invasive Surgery. The team, which included surgeons, anesthetists, nursing staff, and nutritionists, created a standardized clinical pathway for patients. This pathway adhered to ERAS recommendations and published guidelines (5), encompassing over 20 preoperative standard care elements. Overall compliance with 27 items, modified from ERAS guideline 2018, was assessed and expressed as a percentage: the ERAS group was defined as a score of ≥70% and the conventional group was defined as a score of <70% as shown in Table 1.

**Table 1:** ERAS protocol in Hatyai Hospital. Adapted from Gustafsoon et al. (2019) (5), licensed by CC-BY 4.0, to reflect the treatment and nursing care practices at Hatyai Hospital.

Intervention	Recommendation
1. Pre-admission	
1.1 Education and counselling	Preoperative information, stoma care (if any) and postoperative outcomes given
1.2 smoking and alcohol cessation	Stop smoking and alcohol consumption for at least 4 weeks
1.3 Appropriated nutrition support	Patient at risk of malnutrition receive nutritional treatment for a period of at least 7–10 days
1.4 Hb >10 g/dL	Correction of anemia with a targeted concentration of Hb ≥10 g/dL before surgery
2. Pre-operation	
2.1 Pre-anaesthetic medication	Avoided sedatives
2.2 Prevention of nausea and vomiting	Prophylactic use of anti-emetic medications based on Apfel'score
2.3 Antibiotic prophylaxis and skin preparation	Intravenous antibiotic prophylaxis given within 60 min before incision, using chlorhexidine-alcohol-based preparations
2.4 Bowel preparation	Mechanical bowel preparation only used for rectal surgery
2.5 Fluid and electrolytes therapy	Corrected electrolyte, adequate hydration and maintaining euvolemic state before surgery
2.6 Fasting and carbohydrate loading	Allowed eating up until 6 h, and clear fluids including CHO drinks up until 2 h before anaesthesia
2.7 Preoperative multimodal analgesic use	
3. Intra-operation	
3.1 Standard Anaesthetics Protocol	Balanced general anesthesia used
3.2 Thromboprophylaxis	Mechanical thromboprophylaxis (intermittent pneumatic compression) or pharmacological prophylaxis
3.3 Bilateral transversus abdominis plane (TAP) block	Laparoscopic-guided TAP block
3.4 Fluid and electrolytes therapy	Crystalloids <4 mL/kg/h
3.5 Preventing intra-operative hypothermia	Keep BT >36°C
3.6 Surgical access	Minimally invasive surgery or non-midline incision
3.7 Drainage of the peritoneal cavity and pelvis	Avoid routine pelvic and peritoneal drainage
4. Post-operation	
4.1 Nasogastric/orogastric intubation	Postoperative nasogastric/orogastric tubes should be removed before reversal of anesthesia
4.2 Respiratory exercise	Triflow exercise
4.3 Postoperative multimodal analgesic use	Multimodal analgesia, epidural blockade, lidocaine infusion
4.4 Early feeding	Allowing oral intake from the day of surgery
4.5 Early mobilization	Getting out of bed on postoperative day 1
4.6 Fluid and electrolyte therapy	Net “near zero” fluid and electrolyte balance (off removed intravenous fluid on postoperative day 2)
4.7 Urinary drainage	Foley catheter removed on postoperative day 3
4.8 Postoperative glycemic control	Keeping blood sugar ≤200 on postoperative day 0–3
4.9 Prevention of postoperative ileus	Prokinetic drugs given from postoperative day 0

#### Outcomes

The primary outcome of the study was length of postoperative hospital stay measured in days. Secondary outcomes were postoperative complications (e.g., surgical site infection, ileus and anastomotic leakage), re-operation, 30-day readmission, and mortality. Clinical outcomes were evaluated until 30 days postoperatively.

### Statistical analysis

Data were analyzed using R software version 4.4.0, with a significance level set at *α* = 0.05. Continuous variables were compared using the *t*-test; categorical variables were assessed using the chi-square or Fisher's exact test. Logistic regression analysis was performed to identify factors associated with POHS ≤5 days. Receiver operating characteristic (ROC) curves were generated to evaluate the relationship between significant ERAS factors and POHS ≤5 days.

## Results

A total of 140 patients underwent elective laparoscopic colorectal resection at Hatyai Hospital between June 2019 and May 2023: 70 patients in the ERAS group underwent the ERAS program. Table 2 shows relevant patient characteristics and operative parameters. There were no significant differences between the groups regarding age, sex, BMI, disease location, comorbidities, ASA and AJCC staging, or surgical procedures. However, the ERAS group had significantly higher rates of preoperative chemoradiotherapy (CCRT) (52.9% vs. 42.9%; *p* = 0.002) and diverting stoma (38.6% vs. 21.4%; *p* = 0.042).

**Table 2:** Comparison of baseline characteristics between groups.

Patient characteristics	Overall (%)	CC (%)	ERAS (%)	*p*-Value
ERAS compliance score, median (IQR)	18 (13–25)	13 (11–14)	25 (23–26)	<0.001
Age, mean ± SD	62.79 ± 11.3	63 ± 11.9	62.5 ± 10.76	0.807
Male	83 (59.3)	40 (57.1)	43 (61.4)	0.606
Body mass index (kg/m^2^), mean ± SD	22.9 ± 4.0	22.8 ± 4.1	23.0 ± 4.0	0.834
Disease location				
Right-side colon cancer	13 (9.3)	7 (10.0)	6 (8.6)	0.49
Left-side colon cancer	52 (37.1)	29 (41.4)	23 (32.9)	
Rectal cancer	75 (53.6)	34 (48.6)	41 (58.6)	
Upper	12 (8.6)	9 (12.9)	3 (4.3)	0.44
Middle	38 (27.1)	14 (20)	24 (34.3)	
Lower	25 (17.9)	11 (15.7)	14 (20)	
ASA (%)				
Grade I	1 (0.7)	0 (0)	1 (1.4)	0.062
Grade II	63 (45)	44 (62.9)	32 (45.7)	
Grade III	76 (54.3)	26 (37.1)	37 (52.9)	
Comorbidity (%)				
Underlying disease “Yes”	78 (55.7)	33 (47.1)	45 (64.3)	0.061
Hypertension	57 (41.0)	23 (32.9)	34 (48.6)	0.098
Diabetes	35 (25.2)	15 (21.4)	20 (28.6)	0.464
Cardiovascular disease	9 (6.5)	2 (2.9)	7 (10.0)	0.175
Pulmonary disease	7 (5.1)	5 (7.1)	2 (2.9)	0.438
Other	23 (16.5)	10 (14.3)	13 (18.6)	0.675
AJCC staging				
Stage I	14 (10.1)	11 (15.9)	3 (4.3)	0.226
Stage II	38 (27.3)	23 (33.3)	15 (21.4)	
Stage III	82 (9.0)	33 (47.9)	49 (70.0)	
Stage IV	5 (3.6)	2 (2.9)	3 (4.3)	
Surgical procedure				
Right resection (RHC/RC)	18 (12.8)	11 (15.7)	7 (10.0)	0.211
Left resection	6 (4.3)	3 (4.3)	3 (4.3)	
Anterior resection (LAR/ISR)	106 (76.3)	54 (77.1)	52 (74.3)	
APR/TPE	10 (7.2)	2 (2.9)	8 (11.4)	
Preoperative CCRT	55 (39.3)	18 (42.9)	37 (52.9)	0.002
Add diverting stoma	42 (30)	15 (21.4)	27 (38.6)	0.042

LAP, laparoscopic surgery; CC, conventional care; ASA, American Society of Anesthesiologists; RHC, right hemicolectomy; RC, right colectomy; LAR, low anterior resection; ISR, intersphincteric resection; APR, anterior pelvic resection; TPE, total pelvic exenteration; CCRT, concurrent chemoradiotherapy; IQR, interquartile range.

Overall, the study population consisted mainly of elderly patients with ASA grades II–III and showed notable surgical differences between groups. The ERAS group had a higher proportion of diverting stoma cases (52.9%, *n* = 37) compared to the conventional group (42.9%, *n* = 18) (*p* = 0.042), which likely contributed to the shorter median time to initiation of a soft diet.

### Outcomes

Table 3 shows that the ERAS group had a significantly shorter length of hospital stay (7 vs. 5.5 days; *p* < 0.001), POHS (5 vs. 5.5 days; *p* < 0.001), and a quicker transition to a soft diet (4 vs. 3 days; *p* = 0.001). Secondary outcomes are no significant differences between the two groups in anastomotic complications, surgical sites infection, re-intervention rates, or mortality.

**Table 3:** Comparison of results between groups.

Outcome	CC (%)	ERAS (%)	*p*-Value
Operative time (minutes), median (IQR)	240 (180–305)	295 (193–376)	0.042
Estimated blood loss (mL), median (IQR)	100 (50–200)	150 (75–300)	0.160
Intra-operative blood transfusion (units)	8 (11.4)	10 (14.3)	0.801
Removal of drain (days)	5 (4–5)	4 (3–5)	0.006
Remove of rectal tube drain (days)	2 (0–3)	1 (0–2)	0.008
Remove of nasogastric tube (days)	1 (1–1)	0 (0–0)	<0.001
Remove of Foley catheter (days)	2 (1–3)	2 (1–3)	0.917
Date to soft diet	4 (3–4)	3 (3–4)	0.001
Length of hospital stay (days)	7 (6–9)	5.5 (5–7)	<0.001
Postoperative length of hospital stay (days)	5.5 (5–7)	5 (4–6)	<0.001
Overall complications within 30 days	8 (11.4)	8 (11.4)	
Vessel/nerve/ureteric/bladder injury	1 (1.4)	2 (2.9)	1.000
SSI	5 (7.1)	0 (0)	0.058
Anastomosis leakage	4 (5.7)	3 (4.3)	1.000
Ileus	2 (2.9)	2 (2.9)	1.000
Reoperative	4 (5.7)	3 (4.3)	1.000
Readmission in 30 days	2 (2.9)	1 (1.4)	1.000
In hospital mortality	0 (0)	0 (0)	N/A

IQR, interquartile range.

Other significant postoperative advantages in the ERAS group included earlier removal of nasogastric (NG) tubes, Jackson–Pratt (JP) drains, and rectal tubes (all *p* < 0.01). No significant differences were observed between the two groups in terms of overall postoperative complications, including surgical site infections, anastomotic leaks, ileus, re-operation, or in-hospital mortality (*p* > 0.05 for all comparisons).

Multivariate logistic regression identified early JP drain removal, multimodal analgesia, avoidance of NG tubes, early cessation of intravenous fluids, and early urinary catheter removal within postoperative day 3 as independent factors significantly associated with a postoperative stay of ≤5 days (all *p* < 0.05), as shown in Table 4. Full compliance with the ERAS protocol was significantly higher in the ERAS group compared to the conventional group (median compliance score: 25 vs. 13; *p* < 0.001).

**Table 4:** Uni- and multivariate logistic regression analysis of affecting factors (≤5 days).

Effecting factors	Univariate logistic regression		Multivariate logistic regression	
	OR (95% CI)	*p*-Value	OR (95% CI)	*p*-Value
Preoperative CCRT	0.96 (0.88–1.05)	0.337		
Add diverting stoma	1.06 (0.49–2.28)	0.876		
Date of JP drain removal	1.38 (1.14–1.66)	<0.001	1.84 (1.25–2.7)	<0.001
Mechanical bowel preparation	0.29 (0.14–0.61)	<0.001	3.28 (0.55- 19.44)	0.171
Multimodal analgesic use	0.2 (0.09–0.44)	<0.001	0.13 (0.02–0.71)	0.012
No JP drain	1.26 (0.45–3.51)	0.66	32.12 (3.96–260.37)	<0.001
No NG tube	0.19 (0.09,0.41)	<0.001	0.25 (0.09,0.72)	0.008
Off IV within postoperative day 2	0.15 (0.07,0.32)	<0.001	0.26 (0.08,0.84)	0.021
Off Foley catheter within postoperative day 3	0.1 (0.02,0.44)	<0.001	0.2 (0.03,1.21)	0.046

**Figure 2:**
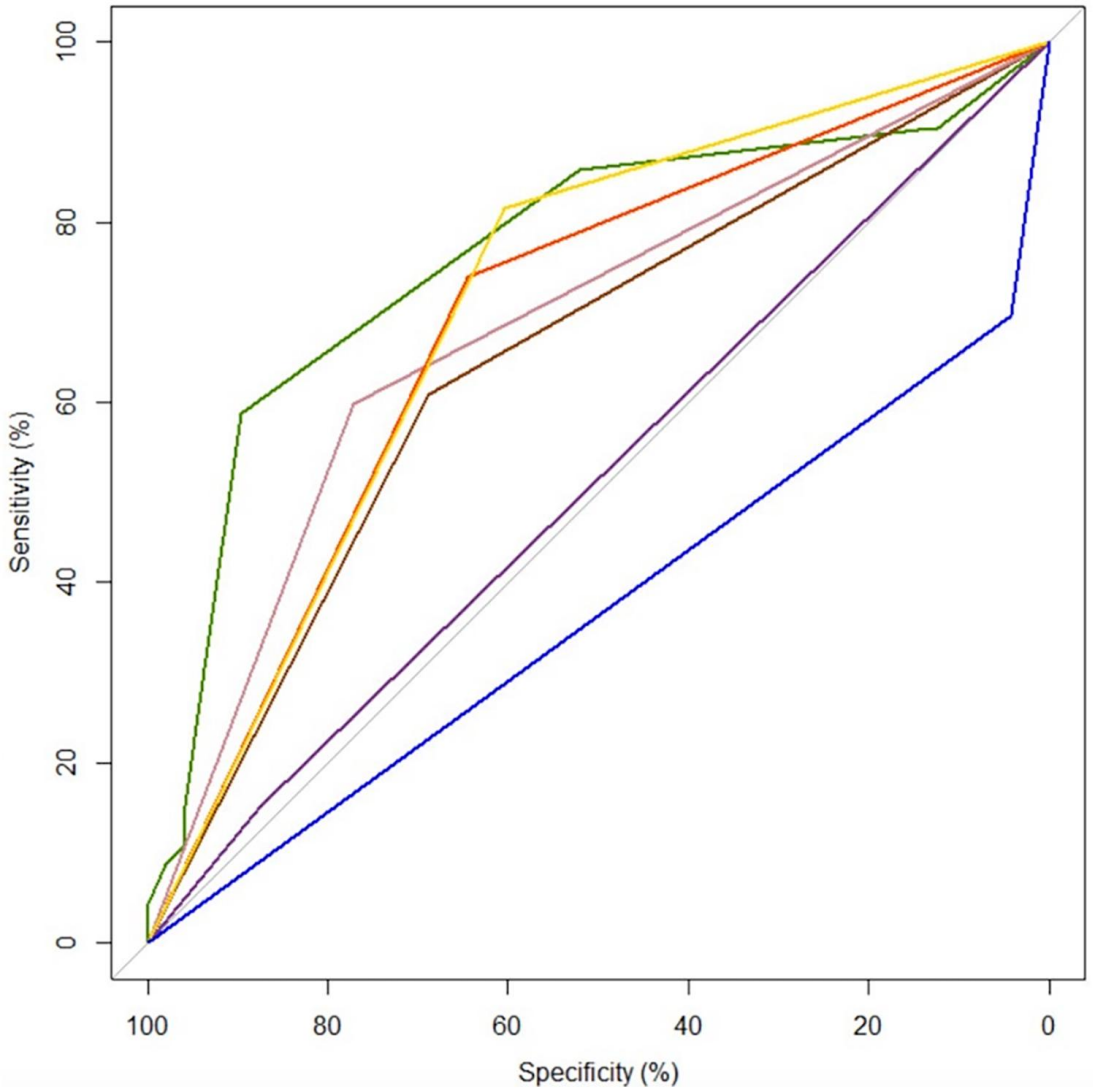
A comparison of the area under the receiver operating characteristic curve (AUROC) of date of JP drain removal 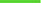, mechanical bowel preparation 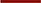, multimodal analgesic use 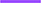, no JP drain 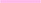, no NG tube 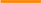, off IV within post operative day 2 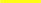 and off Foley catheter within post operative day 3 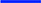 in predicting a postoperative stay of ≤5 days.

The ROC curves of each significant ERAS factor for predicting a postoperative stay of ≤5 days is shown in Figure 2.

The higher rate of diverting stoma formation in the ERAS group may have facilitated earlier oral intake by reducing concerns about anastomotic leakage, which could in turn have influenced other ERAS-related outcomes, such as time to diet and length of stay. In multivariate logistic regression analysis, adherence to the ERAS protocol independently predicted a short hospital stay (≤5 days) (adjusted odds ratio [OR] = 2.46, 95% confidence interval [CI] 1.09–5.56; *p* = 0.030), whereas stoma formation was inversely associated with short hospital stay (adjusted OR = 0.41, 95% CI 0.19–0.89; *p* = 0.024).

## Discussion

This ambispective cohort study demonstrates that the implementation of the ERAS protocol in elective laparoscopic colorectal cancer surgery significantly reduces LOS and POHS without increasing the incidence of complications, readmissions, or mortality. The findings are consistent with the growing body of international literature supporting the efficacy and safety of ERAS pathways in colorectal procedures (5, 8, 9).

The primary outcome, a statistically significant reduction in POHS, suggests that the structured, multidisciplinary approach of ERAS accelerates patient recovery. This effect is likely multifactorial and may be attributed to several key components of the ERAS protocol: early mobilization, early oral feeding, avoidance of NG tubes, timely removal of urinary catheters, optimized pain management through multimodal analgesia, and minimized use of intravenous fluids. Multivariate logistic regression analysis in this study further confirmed that certain ERAS elements, such as early JP drain removal, early discontinuation of intravenous fluids, NG tube avoidance, and multimodal analgesia were significantly associated with shorter hospital stays.

Secondary outcomes, including postoperative complications such as anastomotic leakage, SSI, ileus, and re-operation rates, were not significantly different between the ERAS and conventional care groups. These results underscore that ERAS protocols do not compromise patient safety despite earlier discharge and more aggressive postoperative recovery timelines. Notably, the zero incidence of SSIs in the ERAS group compared to the conventional group (7.1%) may suggest improved perioperative infection control practices, although this did not reach statistical significance.

The readmission rates within 30 days were also comparable between groups, reinforcing the notion that ERAS does not increase post-discharge complications. This is an important consideration for institutions implementing ERAS protocols, particularly in resource-limited settings where healthcare cost containment and bed availability are critical concerns.

A notable finding in the baseline characteristics was the significantly higher proportion of patients receiving preoperative chemoradiotherapy and diverting stomas in the ERAS group. The analysis suggests that the ERAS protocol was successfully implemented in patients with more advanced disease, specifically those with a higher burden of mid-to-lower rectal cancer (54.3% vs. 35.7%) and Stage III disease (70% vs. 47.9%). Crucially, despite this baseline imbalance indicating greater surgical complexity (even without statistical significance in some measures), the ERAS protocol still yielded positive postoperative outcomes. This finding indicates that ERAS is adaptable and can be safely applied even in patients who have undergone intensive preoperative treatment. Although the ERAS group had a higher rate of diverting stoma, the reduction in hospital stay was not solely attributable to stoma-related early feeding but rather to the comprehensive ERAS approach. Moreover, the high compliance rate (≥70%) with ERAS elements in the ERAS group was crucial to the program's success, aligning with prior studies that have shown the relationship between protocol adherence and improved outcome (10, 11). Melliat et al. reported that utilizing a laparoscopic modality alone was insufficient to achieve the short-term outcome benefits of ERAS; compliance to the full clinical protocol was essential. An interesting study by Seow-En et al. demonstrated that the successful implementation of ERAS depends on the surgeon’s experience and that healthcare workers' acceptance towards various components of protocol-based care can directly determine the patient's adherence and sense of confidence in their management (12).

Our study has some strengths. Almost half of the patients were included during a prospective period, allowing for systematic data collection and accurate measurement of outcome events. In addition, all enrolled participants underwent laparoscopic colorectal surgery performed by two surgeons, each with experience in more than 200 laparoscopic colorectal procedures, thereby minimizing surgeon-related confounding factors.

However, several limitations should be acknowledged. First, this study was a non-randomized and time-separated cohort study, which introduces potential biases related to incomplete retrospective data, information bias, and temporal changes in surgical practice. Continuous improvement in surgical skills over time may have influenced the outcomes observed in the later ERAS group. Second, unmeasured confounders—such as socioeconomic status and nutritional status—may have affected the results. In addition, data on long-term oncological outcomes and quality of life were not collected, both of which are important parameters for future prospective studies. Third, the relatively small sample size and the simultaneous implementation of multiple ERAS components may have introduced confounding when assessing the true impact of individual elements.

Despite these limitations, the present study provides valuable insights into the real-world application of ERAS protocols in a regional hospital setting in Thailand. The findings support the broader adoption of ERAS in similar healthcare environments, where resources and budgets may be limited but optimizing clinical outcomes remains a priority. Moreover, this study contributes important evidence from Southeast Asia, a region where data on ERAS implementation remain relatively limited.

## Conclusion

ERAS is a safe and effective approach for elective laparoscopic colorectal cancer surgery, leading to shorter hospital stays, faster recovery, and no increase in complications or readmissions. These short-term benefits may translate into improved long-term survival outcomes, warranting further prospective evaluation across larger patient populations and diverse healthcare settings.

## Data Availability

The original contributions presented in the study are included in the article/Supplementary Material, further inquiries can be directed to the corresponding author.
